# Supplementary material for: Bisimidazolium Salt Glycosyltransferase Inhibitors Suppress Hepatocellular Carcinoma Progression In Vitro and In Vivo
Source: Pharmaceuticals (Basel). 2022 Jun 5;15(6):716. doi: 10.3390/ph15060716 (PMC9229238; doi:10.3390/ph15060716)
Supplement: Supplementary file 1 [file pharmaceuticals-15-00716-s001.zip › pharmaceuticals-1700912-supplementary.pdf]

**Supplementary information**

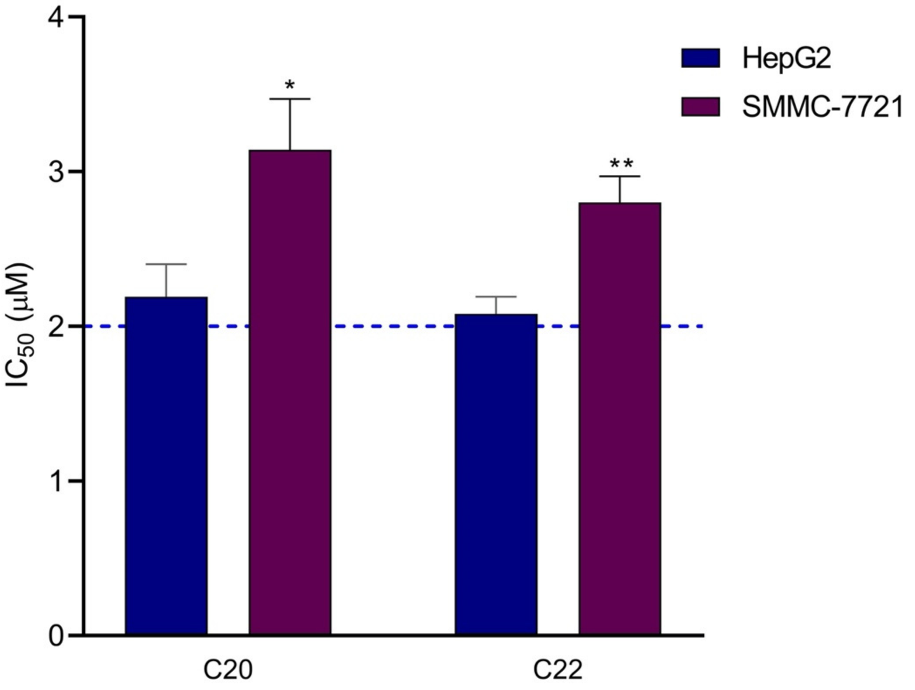


**Figure S1** $IC_{50}$ of C20 and C22 for HepG2 and SMMC-7721 cells. The displayed data correspond to the mean ± SD of three separate experiments. The *p*-value was analyzed by Student *t*-test using GraphPad Prism version 8.00. * $p < 0.05$, ** $p < 0.01$, HepG2 *vs.* SMMC-7721 groups.

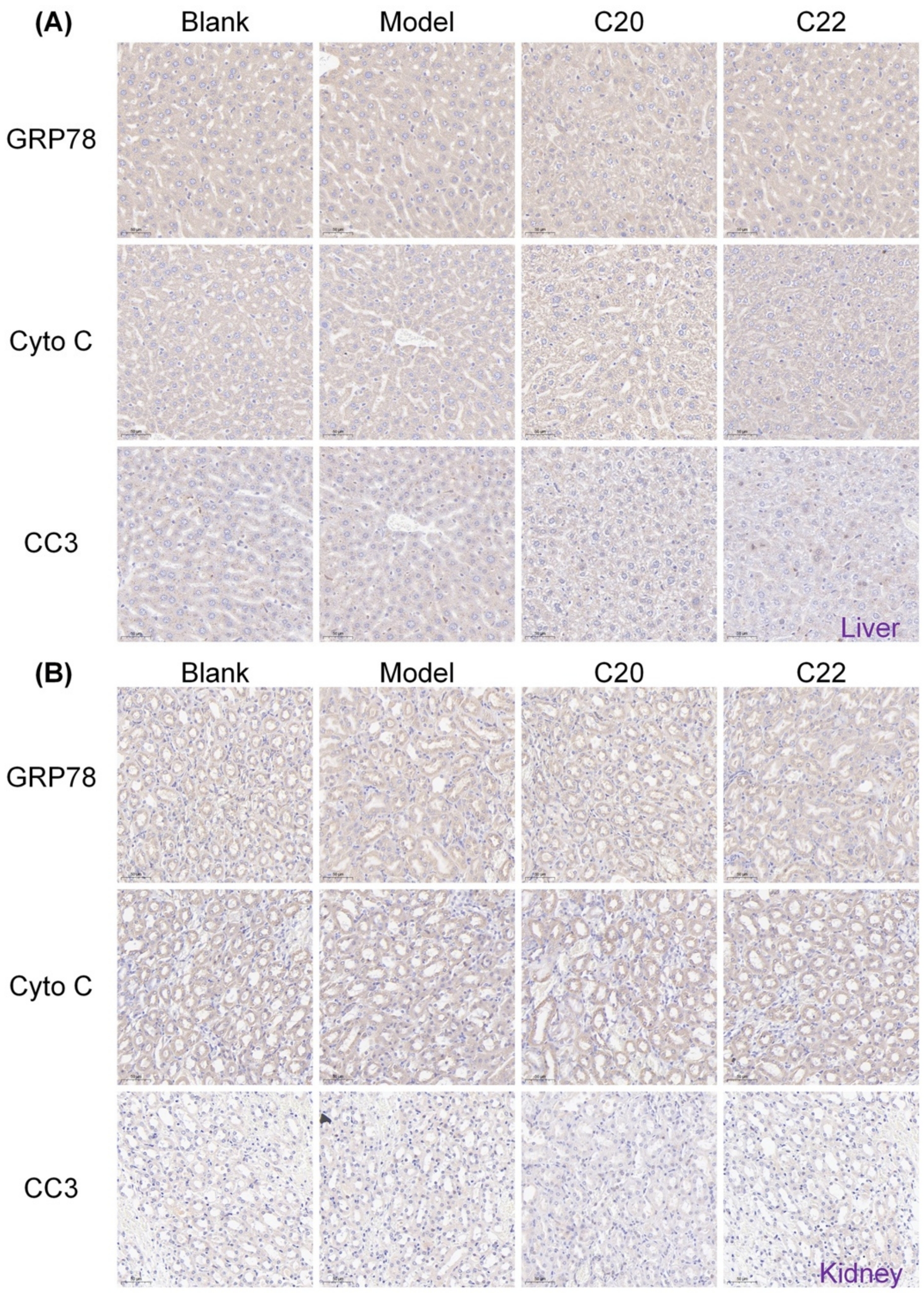


**Figure S2** Immunohistochemistry stain of (A) liver and (B) kidney tissues harvested from the xenograft mice.
